# Robotic bilateral nephrectomy for large polycystic kidney disease

**DOI:** 10.1002/bco2.263

**Published:** 2023-06-24

**Authors:** John M. Masterson, Hanson Zhao, Lior Taich, Aurash Naser‐Tavakolian, Hayley Johnson, Reiad Najjar, Irene K. Kim, Amit Gupta

**Affiliations:** ^1^ Division of Urology Cedars‐Sinai Medical Center Los Angeles California USA; ^2^ Hoag Urology Hoag Hospital Newport Beach California USA; ^3^ Division of Nephrology Cedars‐Sinai Medical Center Los Angeles California USA; ^4^ Department of Surgery Cedars‐Sinai Medical Center Los Angeles California USA; ^5^ Beverly Hills Urology Beverly Hills California USA

**Keywords:** bilateral nephrectomy, end‐stage renal disease, polycystic kidney disease, renal transplant, robotic surgery

## Abstract

**Objective:**

This study aims to describe our technique and review our experience with synchronous robotic bilateral nephrectomy for large kidneys in ADPKD with the da Vinci XI and da Vinci Single Port platforms (Intuitive Surgical, Sunnyvale, CA).

**Materials and Methods:**

We performed a retrospective review of all robotic bilateral nephrectomy cases from January 2020 to present at a high‐volume robotic single centre. Demographic data and perioperative details including preoperative CT scans, indication for nephrectomy and renal function were collected. We also collected post‐op course data and final specimen data details.

**Results:**

Fourteen cases were included. Patient demographics, indications for surgery and specimen data are outlined in Table 1. The largest kidney removed has a measurement of 32 cm in the largest dimension on preoperative imaging. Median operating time from incision to closure was 299 min (IQR 260, 339). Median estimated blood loss was 75 cc (IQR 50, 187.5). Two patients were transfused intraoperatively. Median pre‐ and post‐operative Hgb was 11.0 and 9.6, respectively. Median length of stay was 3 days (IQR 2, 3.5). There were no intraoperative complications and no open conversions. Post‐operative complications included one incisional hematoma and one superficial wound infection. One patient was admitted to the surgical ICU post operatively for ventilatory support. Two patients were readmitted within 30 days of surgery.

**Conclusion:**

The robotic approach to bilateral native nephrectomy for ADPKD should be considered when native nephrectomies are indicated. The operative times and outcomes are favourable compared with prior series, and this technique works even for very large kidneys.

## 1 INTRODUCTION

Autosomal dominant polycystic kidney disease (ADPKD) is the most common hereditary kidney disorder with an estimated prevalence of one in 1000 to one in 2500 individuals.^1^ ADPKD is characterized by the progressive development and growth of cysts in the renal parenchyma, resulting in renal deterioration. Approximately 50% of patients with ADPKD have end stage renal disease (ESRD) by age 60.^2^

Indications for native nephrectomy in patients with ADPKD include intractable pain, haemorrhage, early satiety, recurrent infections, infected stones suspicion for renal cancer and need to create space for renal transplant allograft. Historically, open bilateral native nephrectomy has been a highly morbid and even fatal procedure. Early series have demonstrated major complication rates of up to 38% and mortality rates of 3%–5%.^3^, ^4^ More modern series report estimated blood loss (EBL) in open nephrectomy ranges from 723 to 1454 cc, leading to high rates of blood transfusion, which is of particular concern in transplantation due to risk of broad sensitization to human leukocyte antigen (HLA) phenotypes and subsequent graft rejection.^5^ Length of stay (LOS) in open nephrectomy ranges from 7 to 10 days, and complications requiring a second surgery occur in 20%–60% of patients—most commonly incisional hernia repair of the xiphoid to pubis midline incision.^5^, ^6^ Because of the risks associated with open bilateral native nephrectomy, the optimal timing and necessity of native nephrectomy for patients with ADPKD has been debated, with some arguing that open native nephrectomy in ADPKD should be avoided as much as possible.^1^, ^7^, ^8^, ^9^, ^10^

Minimally invasive laparoscopic techniques for synchronous bilateral nephrectomy have been described in an attempt to reduce the morbidity associated with the procedure.^11^ However, there have been shortcomings with the laparoscopic approach—especially for larger polycystic kidneys—such as prolonged operative time and open conversion rates as high as 22%.^12^, ^13^, ^14^ Interestingly, despite the rapid adoption of the robotic platform among urologic surgeons even in challenging clinical scenarios such as redo partial nephrectomy and partial nephrectomy for endophytic tumours, there has been only a few descriptions regarding a robotic approach to ADPKD.^15^, ^16^, ^17^, ^18^ In this study, we discuss our technique and review our experience with synchronous robotic bilateral nephrectomy for large kidneys in ADPKD using the da Vinci Xi and da Vinci Single Port platforms (Intuitive Surgical, Sunnyvale, CA).

## 2 MATERIALS AND METHODS

After the Institutional Review Board approval was obtained, we performed a retrospective review of all robotic bilateral nephrectomy for ADPKD cases at Cedars‐Sinai Medical Center from January 2020 to present performed by a single surgeon (Amit Gupta, MD, MPH). All such cases were included; none were excluded. We collected demographic details about the patients and reviewed perioperative details including preoperative CT scans and indication for nephrectomy. We specifically recorded patient age at surgery, height, BMI, pre‐operative haemoglobin (Hgb), largest kidney dimension and ellipsoid volume on preoperative CT and whether patients were on peritoneal dialysis preoperatively, as this is an important logistical consideration for post‐operative conversion to haemodialysis. We also collected details on the intra‐ and post‐operative course including robotic platform used, total console time, total case time, EBL, post‐operative Hgb, LOS, surgical complications based on Clavien–Dindo classification and 30‐day readmission.^19^ In this series, the da Vinci Xi platform (Intuitive Surgical, Sunnyvale, CA) was used for 13 cases, and the da Vinci SP platform (Intuitive Surgical, Sunnyvale, CA) was used for one case.

### 2.1 Da Vinci Xi technique

#### 2.1.1 Positioning and port placement

The patient is positioned in a modified flank position similar to that of a radical or partial nephrectomy. We use a Veress needle to initiate insufflation. Once the peritoneum is insufflated to 12–15 mmHg, we then place four 8 mm robotic ports in a linear configuration along the midclavicular line. The most cephalad port is placed right below the ribcage and the most caudal port in the lower quadrant. The remaining middle two ports are equally spaced out in between. A 12 mm AirSeal (Conmed, Utica, NY) is then placed slightly cephalad to the umbilicus. For the left nephrectomy, our robotic instruments from cephalad to caudad are the Fenestrated Bipolar, a 30‐degree camera, monopolar scissors and ProGrasp. For the right nephrectomy, our robotic instruments from cephalad to caudad are the monopolar scissors, a 30‐degree camera, the Fenestrated Bipolar and ProGrasp. The surgery can be started with either side first.

#### 2.1.2 Surgical technique

On the left side, the procedure is initiated in usual fashion. The white line of Toldt is incised, and the descending colon is reflected medially along with the spleen and pancreas. We then enter Gerota's fascia underneath the lower pole of the kidney to identify and divide the gonadal vein and ureter. The fourth arm is used to elevate the kidney above the psoas muscle. At this point, we often switch to a 30° up configuration. The hilum is then dissected out and stapled, and the adrenal gland is spared. The remaining superior, lateral and inferior attachments are then divided. The fourth arm is critical throughout the case and helps manipulate the kidney to provide tension in all directions. Once the kidney is fully mobilized, we place it into the lower abdomen and pelvis. In our experience, we never had to remove one kidney for the purpose of better visualization or mobilization of the contralateral side.

**FIGURE 1:**
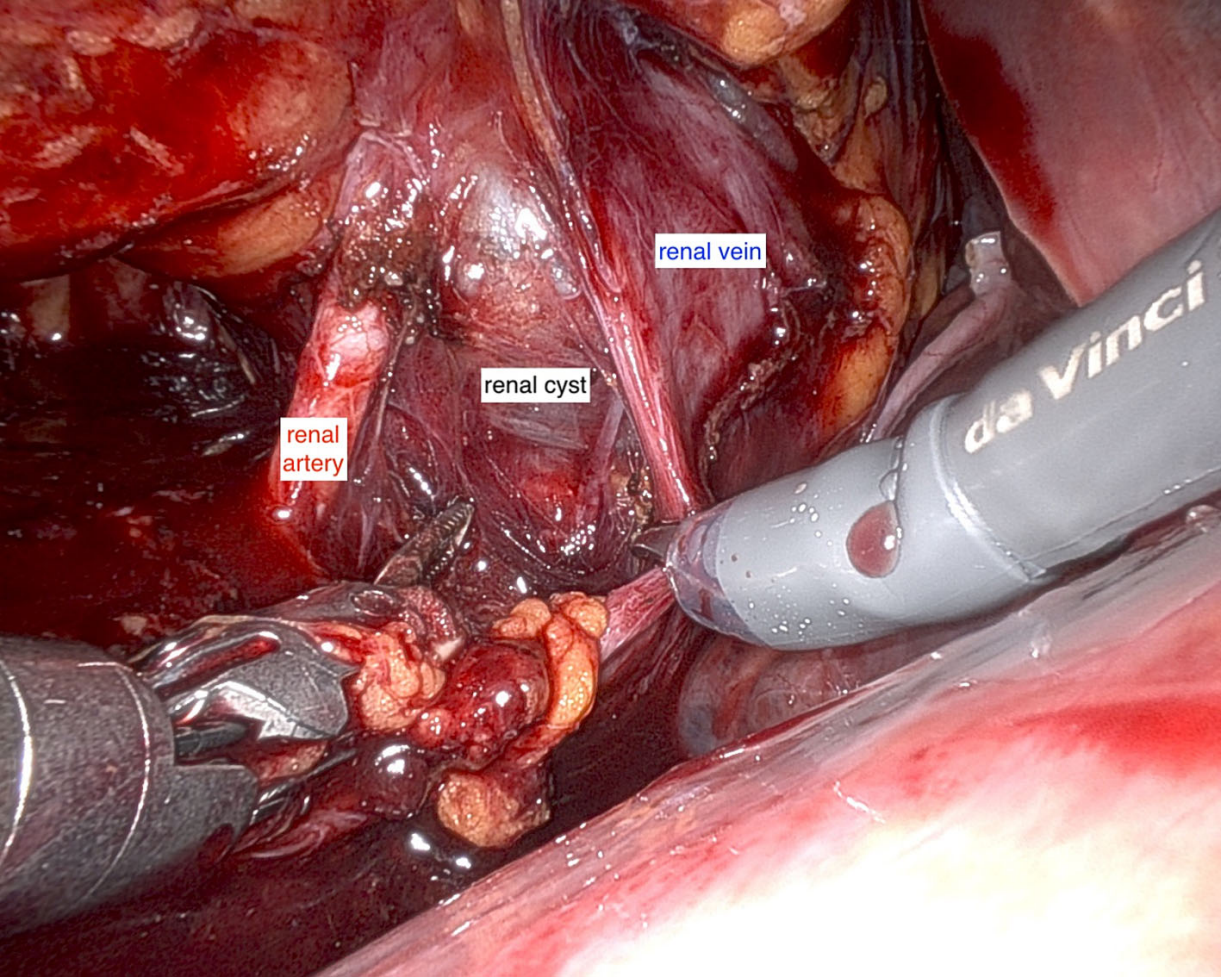
Right‐sided renal hilar dissection with large cyst distorting the renal hilum and flattening the right renal vein and the inferior vena cava (IVC).

**FIGURE 2:**
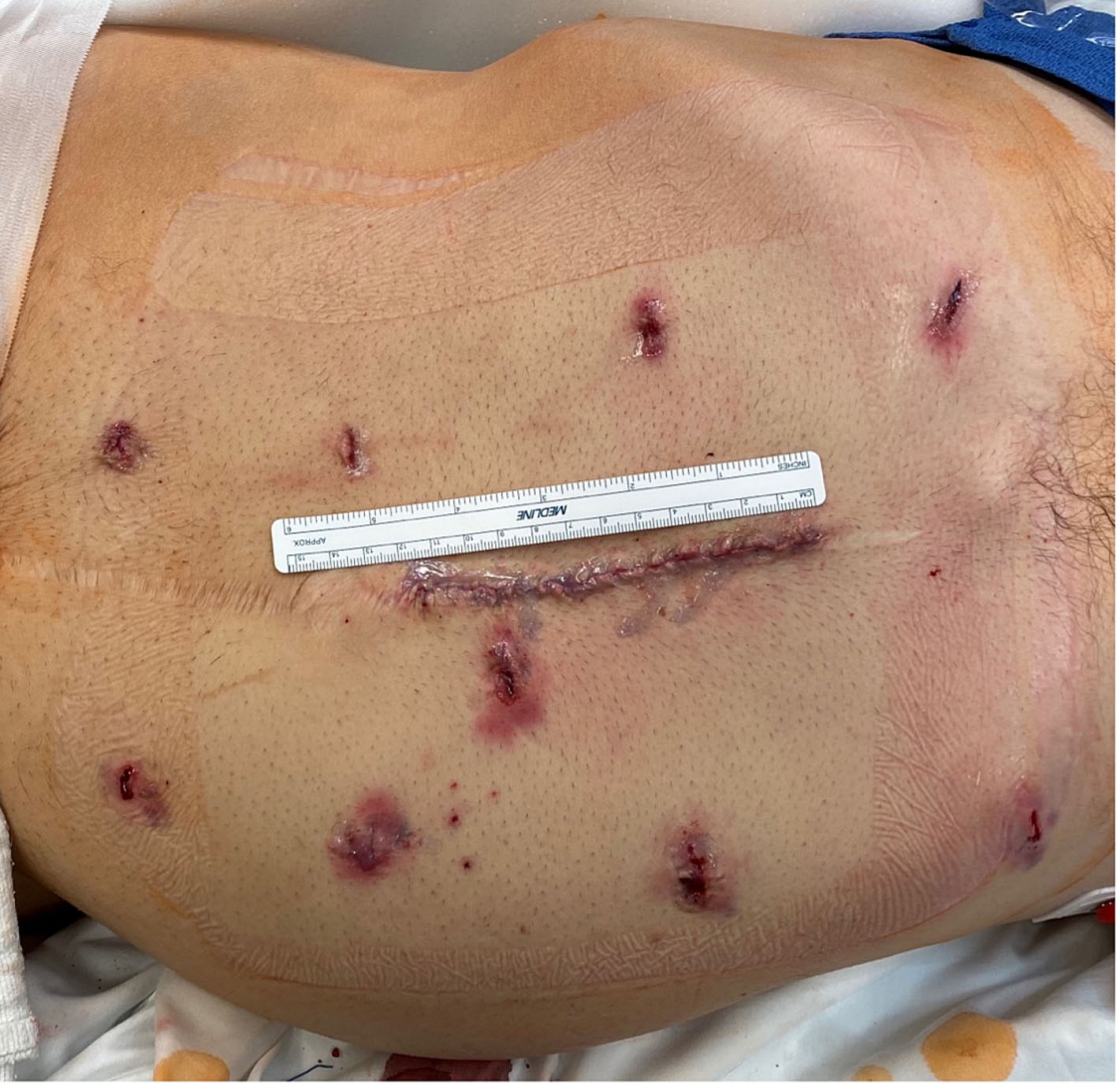
Closed port sites and midline extraction site with Xi platform.

At this point, the robot is undocked, the instruments and ports are removed and the four robotic ports are closed with Monocryl sutures and skin glue. We place a Tegaderm (3 M, St. Paul, MN) over the assistant port. The robotic cords, camera and AirSeal tubing are kept sterile on a separate mayo stand. The drapes are removed, and the patient is repositioned for the contralateral side and prepped and draped in the usual fashion. The Tegaderm is removed, and the AirSeal port is replaced. Pneumoperitoneum is easily re‐established through the assistant port. Four more robotic ports are placed along the right midclavicular line to mirror those on the left with the instruments as previously described. On the right side, we have not had to use a liver retractor routinely unless the liver is extremely large. The right nephrectomy is carried out starting with the reflection of the ascending colon and the duodenum. With very large kidneys, the inferior vena cava is often displaced and adherent to the medial surface of the kidney, so care must be taken to identify and dissect the cava. Occasionally, cysts from the kidney may crowd and distort the renal hilum (Figure 1). The remainder of the dissection mirrors the left side. The adrenal gland is spared. Once the kidney is freed, a transverse abdominis plane block is performed under visual guidance. The midline assistant port is extended superiorly to remove both specimens, and all incisions are closed with Monocryl sutures (Figure 2).

### 2.2 Da Vinci SP technique

The positioning is similar to the da Vinci Xi technique. We airdock the SP trocar through a GelPoint (Applied Medical, Rancho Santa Margarita, CA) on a paramedian incision. A 10 mm AirSeal (Conmed, Utica, NY) is placed along the midline. We use monopolar scissors and two Fenestrated Bipolars so that we can easily alternate between retraction and cautery. We initiate the case with the camera at the 12 o'clock position. Once we start dissecting on the psoas toward the hilum, we rotate the port and arms 180° so that the camera is at the 6 o'clock position, and the kidney can be lifted freely. The rest of the dissection is carried out as previously described. If the anatomy allows, we staple the inferior and lateral attachments to expedite the dissection. The kidneys are removed by extending the midline incision at the end of the case.

#### 2.2.1 Post‐operative details

The patients are closely monitored on the surgical floors. As majority of these patients were on dialysis at the time of their surgeries, the nephrology teams were consulted to manage inpatient dialysis. The patients are discharged and follow up in 2–3 weeks.

## 3 RESULTS

**FIGURE 3:**
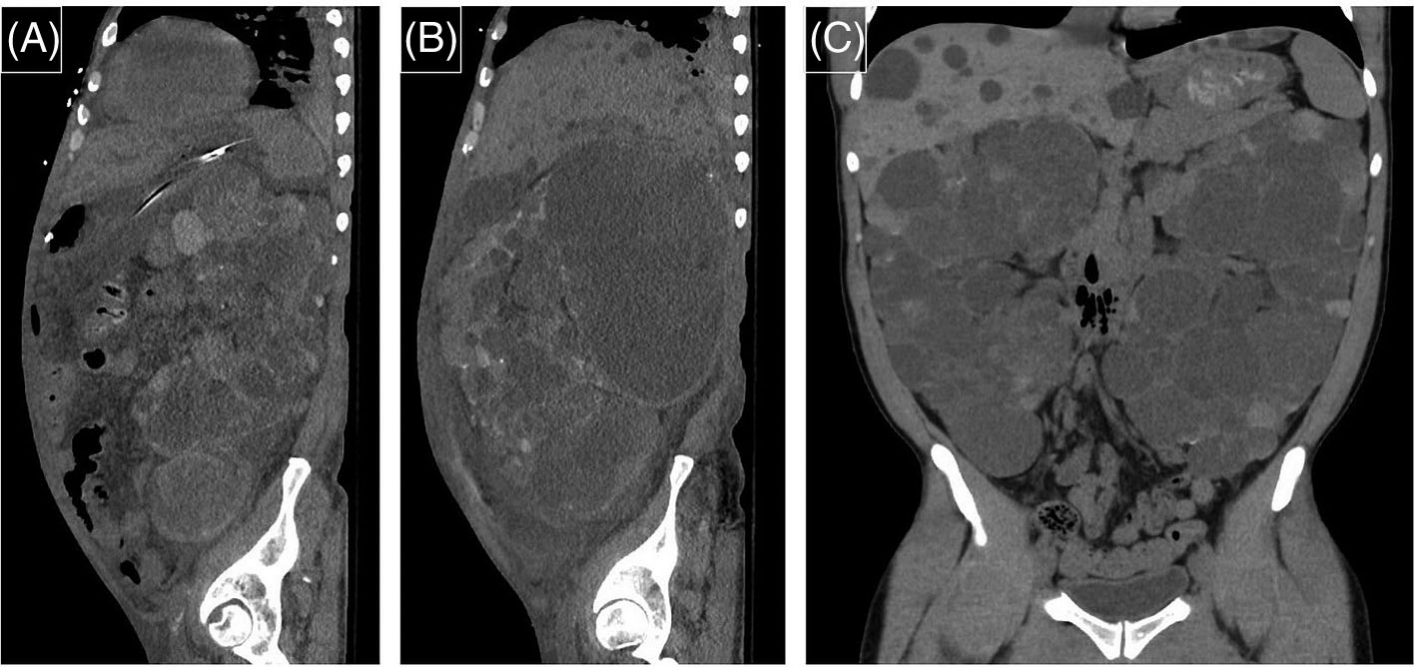
Preoperative computed tomography (CT) images of polycystic kidneys: (A) largest right kidney removed 29.3 cm craniocaudally; (B) largest left kidney removed 31.8 cm craniocaudally and (C) bilateral kidneys cross midline and nearly fill the abdominal cavity.

A total of 14 cases of synchronous robotic bilateral nephrectomies for APCKD were performed from January 2020 to the time of publication—summarized preoperative data is displayed in Table 1 and detailed preoperative data is displayed in Table 2. All patients referred to the surgeon (AG) underwent robotic surgery and no open nephrectomies were performed by the surgeon or referred to another surgeon for open nephrectomy. There were nine female and five male patients. The median age was 48 years (IQR 44, 56). The median BMI was 26.3 kg/m (IQR 23.7, 31.8). Indications for bilateral nephrectomy included creating room for a kidney transplant (*n* = 9), abdominal discomfort (*n* = 7), gross hematuria (*n* = 2) and recurrent renal abscess (*n* = 1). Thirteen cases were performed before transplant, one case was performed post‐transplant in a patient who experienced graft failure after 14 years, and one case was performed after transplant with a well‐functioning graft. Based on preoperative computed tomography (CT) imaging, right and left kidney size ranged from 14.3 to 29.3 cm and 15.6 to 31.8 cm, respectively, based on the largest measured dimension (Figure 3); the calculated ellipsoid volume of right and left kidneys ranged from 774.0 to 7600.3 cm^3^ and 1051.5 to 3778.9 cm^3^, respectively (Table 1).^1^ Three patients who were on peritoneal dialysis were converted to haemodialysis pre‐operatively, one of whom experienced an extended inpatient stay because of her temporary haemodialysis catheter causing symptomatic tachycardia. They resumed peritoneal dialysis 4–6 weeks after surgery.

**TABLE 1:** Summarized population characteristics and surgical outcomes.

Population Characteristics	*N* (%)
Total subjects	14 (100%)
Female	9 (64.3%)
Male	5 (35.7%)
Age (median, IQR)	48 (44, 56)
BMI (median, IQR)	26.3 (23.7, 31.8)
Indication for surgery	
To create space for transplant	9 (69.2%)
Symptomatic (abdominal distention, hematuria)	6 (46.2%)
Recurrent pyelonephritis	1 (9.1%)
Surgical details	
Multi port, Xi	13 (92.9%)
Single port, Sp	1 (7.1%)
Total case time in minutes (median, IQR)	299 (253, 357)
Total console time in minutes (median, IQR)	189 (161, 215.5)
Pre‐operative Hgb in g/dL (median, IQR)	11 (9.9, 11.8)
Post‐operative Hgb in g/dL (median, IQR)	9.6 (9.1, 10.4)
Estimated blood loss in cc (median, IQR)	75 (50, 187.5)
Length of stay in days (median, IQR)	3 (2, 3.5)
Complications	
Blood transfusion	2 (18.2%)
30‐day readmit	2 (18.2%)
ICU stay	1 (9.1%)
Abdominal wall hematoma	1 (9.1%)
Surgical wound infection	1 (9.1%)
Pathology	
Benign	13 (100%)
Malignant	0 (0%)
Largest kidney dimension on preop CT (cm)	
Right kidney (range)	14.3–29.3
Left kidney (range)	15.6–31.8
Ellipsoid kidney volume (cm3)	
Right kidney (range)	613.1–5478.8
Left kidney (range)	771.2–3421.0

*Note*: One patient transfused with autologous blood and one patient with baseline anaemia preoperatively (Hgb 8.8 g/dL).

Abbreviations: BMI, body mass index; CT, computed tomography; ICU, intensive care unit; IQR, interquartile range.

**TABLE 2:** Detailed patient demographics and pre‐operative considerations.

Case	Gender	Age (years)	Height (meters)	BMI	Indication for surgery	CT largest kidney dimension (cm^3^)		Ellipsoid renal volume calculation (cm^3^)		Peritoneal dialysis pre‐operatively
						Right	Left	Right	Left	
1	F	38	1.63	32	Room for transplant, flank pain, abdominal pain/distension, gross hematuria	23.8	30	2532.1	4754.2	No
2	M	58	1.75	25	Room for transplant	25	21.2	3176.4	2636.6	Yes
3	M	46	1.96	24	Room for transplant, gross hematuria	24.3	24.3	4044.1	3792.8	No
4	F	44	1.5	38	Oral intake intolerance, bloating, abdominal distension	18.8	18.9	1104.7	2162.2	No
5	M	34	1.68	22	Abdominal distension, discomfort	22.8	23.7	3524.8	3285.7	No
6	M	50	1.65	23	Room for transplant	27.8	23.8	7267.8	3161.6	No
7	F	48	1.68	33	Recurrent pyelonephritis/renal abscess	15.2	15.6	672.7	1652.0	No
8	F	60	1.5	33	Room for transplant	23.5	19.4	4531.8	3329.8	No
9	F	48	1.22	27	Room for transplant	19.8	21	1698.6	1925.4	Yes
10	M	68	1.68	23	Room for transplant	18.3	16.7	1034.1	1190.0	No
11	F	56	1.63	28	Flank pain	14.3	17.6	1134.8	710.0	No
12	F	43	1.68	26	Room for Transplant, abdominal pain, abdominal distension	27	25	3242.8	3247.8	Yes
13	F	56	1.78	24	Room for transplant	25	26	1782.5	2242.7	No
14	F	57	1.68	22	Oral intake intolerance, bloating, abdominal distension	22	22.7	1327.0	1409.5	No

Abbreviations: BMI, body mass index; CT, computed tomography.

Summarized preoperative data is displayed in Table 1 and detailed operative and post‐operative data is displayed in Table 3. Median total robotic operating time combining dock to undock time for each kidney was 189 min (IQR 161, 215.5), while median total operating time from incision to close was 299 min (IQR 260, 338.5). Median EBL was 75 cc (IQR 50, 187.5). Two patients were transfused intraoperatively, one with autologous blood due to intraoperative blood loss and the second due to baseline anaemia with a low preoperative Hgb of 8.8 g/dl. The median preoperative and postoperative Hgb were 11.0 (IQR 9.9, 11.8) and 9.6 g/dL (IQR 9.1, 10.4), respectively. There were no intraoperative complications and conversions to open procedure. The median LOS was 3 days (IQR 2.0, 3.5). One patient—the same patient who required autologous blood transfusion intraoperatively—was kept intubated post‐operatively in the surgical intensive care unit overnight, but was extubated and downgraded on post‐op day one, did not require pressor support and was discharged home on post‐op Day 2. In the post‐operative setting, two patients were readmitted within 30 days; one patient developed a subcutaneous hematoma below the midline incision, which was drained at bedside representing a Clavien–Dindo grade I complication. Another patient developed a superficial wound infection, which was managed conservatively with antibiotics representing a Clavien–Dindo grade I complication. There were no other complications noted. The pathology for all kidneys was benign. Four patients successfully underwent a kidney transplantation at a median of 95 days (IQR 81 to 108) after bilateral nephrectomy.

**TABLE 3:** Intra‐operative and post‐operative details.

Case	Platform	Console time	EBL (cc)	Transfusion	Pre‐op Hgb	Post‐op Hgb	LOS (days)	Complications	30‐day readmission due to surgical complication
1	Xi	3:10	100	No	10	9.5	3	None	No
2	Xi	2:14	100	No	10.4	9.5	3	None	No
3	Xi	3:05	200	No	12.6	12.4	1.5	None	No
4	Xi	2:41	50	No	9.5	8.3	4	Wound seroma	No
5	Xi	3:08	150	No	11.3	9.7	5^a^	None	No
6	Xi	4:49	700	Yes^b^	12.9	10.4	2	None	No
7	SP	3:38	400	No	8.8	9.2	9^c^	Wound infection	Yes
8	Xi	2:34	20	No	11.8	10.4	2	None	No
9	Xi	3:36	30	No	9.8	10.6	3	None	No
10	Xi	2:17	50	No	11.9	9.1	3	None	No
11	Xi	3:34	50	No	11.5	11.6	2	None	No
12	Xi	4:22	200	No	10.6	8.6	6^d^	None	No
13	Xi	2:44	25	No	8.6	7.9	3	None	No
14	Xi	3:11	50	No	11.6	10.3	2	None	No

Abbreviations: EBL, estimated blood loss; Hgb, haemoglobin; LOS, length of stay.

^a^ Extended LOS due to scheduling issues related to outpatient dialysis.

^b^ Autologous blood transfusion.

^c^ Extended LOS due to acute on chronic pain control issues.

^d^ Extended LOS due to issues with temporary haemodialysis tunnelled catheter placement.

## 4 DISCUSSION

APCKD is a common cause of renal failure, and up to 25% of patients with APCKD will undergo native nephrectomy.^20^ Open bilateral native nephrectomy has historically been a highly morbid procedure, and because of the potential risks associated with the operation, the timing and need for removal of both native kidneys has been debated.^1^, ^7^, ^8^, ^9^, ^10^ Several series have demonstrated success with laparoscopic removal of large polycystic kidneys; however, robotic approaches to this procedure have only recently been described. In this study, we describe our technique for pre‐transplant synchronous bilateral robotic nephrectomy for ADPKD with the da Vinci Xi and SP robotic systems. We present a series of 14 consecutive patients who successfully underwent this procedure at our institution with a median LOS of 3 days. This robotic approach appears to be safe and well tolerated even for extremely large polycystic kidneys. One patient had a prior exploratory laparotomy incision extending from xiphoid to the pubis for peritonitis because of hollow viscus perforation, 18 months prior. Even in this patient, we were able to achieve robotic bilateral nephrectomy with no complications (Figure 2).

Our robotic technique demonstrated several marked advantages over traditional open bilateral native nephrectomy. Kramer et al. updated their institutional experience over 8 years with combined open bilateral native nephrectomy and living donor renal transplant. Mean operating time for the nephrectomy portion in their series was 191 min compared with our median cumulative dock‐to‐undock time of 188 min.^5^ LOS for open nephrectomy in the Kramer series was 7.2 days—which did not improve over their 8‐year experience—compared with our median LOS of 3 days.^5^ Ismail et al. reported complications requiring a separate operation in seven of their 11 patients who underwent open bilateral nephrectomy for ADPKD; five of whom experienced incisional hernias requiring repair while our series demonstrated no incisional hernias. Mean EBL for open nephrectomy in the Ismail et al. series was 1635 cc, while Kramer et al. reported a mean EBL of 723 cc—which was an improvement over the mean EBL of 1454 cc in their original series 8 years prior—resulting in a 90% transfusion rate at an average of 3.3 units of blood per patient.^5^, ^6^ Our technique resulted in transfusion of two patients: one with two units of autologous blood and the other with a unit of packed red blood cells in the setting of baseline anaemia with a pre‐operative Hgb of 8.8 g/dL. Transfusion requirement is an important consideration in the pre‐transplant native nephrectomy setting given the risk of broad HLA sensitization associated with blood transfusion. Sensitization is associated with difficulty finding a well HLA‐matched donor, longer transplant wait times, more rejection episodes and inferior long‐term graft and patient survival, highlighting the importance of minimal intraoperative blood loss.^21^, ^22^, ^23^

Minimally invasive techniques prior to introduction of the da Vinci robotic platforms have helped to address this issue of significant intraoperative blood loss and morbidity related to open nephrectomy. The earliest series of laparoscopic bilateral nephrectomy for ADPKD reported a mean EBL of only 85 cc but experienced prolonged operating times of approximately 470 min.^11^ Subsequent series have reported both modest and significant improvement on this figure while maintaining similarly improved EBL compared with the open technique. Desai et al. reported a mean surgical time of 214 min with a mean EBL of 169 cc among 12 patients undergoing laparoscopic nephrectomy for massive polycystic kidneys.^13^ Gill et al. and Dunn et al. reported an average operative time of 264 and 378 min and mean EBL of 150 and 153 cc among their respective series of 10 and 11 patients undergoing laparoscopic nephrectomy for ADPKD.^24^, ^25^ Although our technique requires that the patient be repositioned, prepped and draped, this generally does not appear to add a significant amount of time. Our median operative time including repositioning and reprepping was 299 min, which is favourable compared with these other minimally invasive series. Laparoscopic series also report varying rates of conversion to open nephrectomy, ranging from 0% to 22%.^12^, ^13^, ^14^ Despite the large size of many of our polycystic kidneys, no case required conversion to open nephrectomy. These laparoscopic series similarly demonstrate minimal risk of incisional hernia and decreased LOS compared with open nephrectomy.^11^, ^13^, ^24^, ^25^

Gurung et al. recently reported their series of seven patients with ADPKD who underwent robotic bilateral nephrectomy with the da Vinci Xi platform.^18^ These patients were positioned supine with the table tilted in Trendelenburg and laterally. The authors used a transverse port arrangement at the level of the umbilicus for the entire case. When transitioning to the contralateral side, the robot just needs to be redocked with the table titled the opposite direction. This has the added advantage of reducing the number of ports and minimizes the time it takes to reposition and re‐prep the patient. In their series, the median operating room time was 388 min, the median EBL was 200 cc with no blood transfusions, the median LOS was 3 days and there were no major complications.^18^ This technique appears to work well for smaller polycystic kidneys, as the representative images in their series appear much smaller than ours. The indications for nephrectomy were for pain, haemorrhage and renal mass, and all had prior kidney transplants.

Importantly, we believe that optimizing port placement for each side allows for removal of kidneys of any size. We also avoid the need to intentionally puncture cysts during the case, which may cause unnecessary spillage of potentially infectious and irritating contents that may lead to ileus and peritoneal irritation similar to chemical peritonitis. As this port placement and operative approach is familiar to most urologists, it can minimize complications. In our series, there were no intraoperative events. The only two, Clavien–Dindo grade I complications occurred post‐operatively: one patient developed a wound hematoma, which was drained, and another patient developed a superficial wound infection, which was managed with antibiotics—this patient had a history of recurrent pyelonephritis and renal abscesses.

Ultimately, the timing and necessity of bilateral native nephrectomies for ADPKD should be catered to each individual patient. In this series, we demonstrate that robotic bilateral native nephrectomy for ADPKD is safe and effective even for large polycystic kidneys. Four patients successfully underwent an extraperitoneal kidney transplant at a median 95 days after native nephrectomy. The limitations of our study include the small sample size, retrospective design and lack of a comparison arm. However, to our knowledge, this is the largest robotic series on bilateral nephrectomy for ADPKD and the first to describe the procedure with the da Vinci SP platform.

## 5 CONCLUSION

The robotic approach to bilateral native nephrectomy for ADPKD is effective for large kidneys and should be considered when indicated. Robotic bilateral nephrectomy offers a minimally invasive procedure with several important advantages over open nephrectomy including decreased blood loss, LOS and post‐operative complication rate.

## AUTHOR CONTRIBUTIONS

**John M. Masterson:** Methodology; formal analysis; data curation; writing original draft. **Hanson Zhao:** Conceptualization; methodology; writing—review and editing. **Lior Taich:** Conceptualization; resources. **Aurash Naser‐Tavakolian:** Conceptualization; methodology; writing—review and editing. **Hayley Johnson:** Conceptualization; methodology; writing—review and editing. **Reiad Najjar:** Writing—review and editing. **Irene K. Kim:** Writing—review and editing. **Amit Gupta:** Methodology; formal analysis; data curation; writing original draft; writing—review and editing; supervision.

## CONFLICTS OF INTEREST STATEMENT

All authors have no conflicts of interest to disclose.
